# Every Child on the Map: A Theory of Change Framework for Improving Childhood Immunization Coverage and Equity Using Geospatial Data and Technologies

**DOI:** 10.2196/29759

**Published:** 2021-08-03

**Authors:** Sarah Cunard Chaney, Patricia Mechael, Nay Myo Thu, Mamadou S Diallo, Carine Gachen

**Affiliations:** 1 HealthEnabled Cape Town South Africa; 2 UNICEF Juba; 3 Data and Analytics Unit Department of Analysis, Planning & Monitoring UNICEF New York, NY United States; 4 Gavi, the Vaccine Alliance Health Information Systems and Digital Health Information Geneva Switzerland

**Keywords:** geospatial data, immunization, health information systems, service delivery, equity mapping, theory, framework, children, immunization, vaccine, equity, geospatial, data, outcome, coverage, low- and middle-income, LMIC

## Abstract

The effective use of geospatial data and technologies to collect, manage, analyze, model, and visualize geographic data has great potential to improve data-driven decision-making for immunization programs. This article presents a theory of change for the use of geospatial technologies for immunization programming—a framework to illustrate the ways in which geospatial data and technologies can contribute to improved immunization outcomes and have a positive impact on childhood immunization coverage rates in low- and middle-income countries. The theory of change is the result of a review of the state of the evidence and literature; consultation with implementers, donors, and immunization and geospatial technology experts; and a review of country-level implementation experiences. The framework illustrates how the effective use of geospatial data and technologies can help immunization programs realize improvements in the number of children immunized by producing reliable estimates of target populations, identifying chronically missed settlements and locations with the highest number of zero-dose and under-immunized children, and guiding immunization managers with solutions to optimize resource distribution and location of health services. Through these direct effects on service delivery, geospatial data and technologies can contribute to the strengthening of the overall health system with equity in immunization coverage. Recent implementation of integrated geospatial data and technologies for the immunization program in Myanmar demonstrate the process that countries may experience on the path to achieving lasting systematic improvements. The theory of change presented here may serve as a guide for country program managers, implementers, donors, and other stakeholders to better understand how geospatial tools can support immunization programs and facilitate integrated service planning and equitable delivery through the unifying role of geography and geospatial data.

## Introduction

Maps are powerful tools for public health decision-makers to better understand the relationship between the location of populations and health system resources, indicators or predictors of health status, and their patterns over space and time. The visual power of the map is aided by modern advances in technology, computing, and handheld devices that can record the location of any place on the earth and transmit geospatial data for analysis, sharing, and use. The use of geography to analyze patterns of disease, distribution of populations, and inventories and locations of health services come together to create a catalyst for improving health systems.

Immunization programs in low- and middle-income countries are beginning to harness digital maps and geospatial data to display and analyze complex information for program improvements [1-6]. The effective use of geospatial data can show program managers which locations have not received adequate immunization services, provide more accurate denominators, and inform what vaccination delivery strategies should be used to optimize coverage and equity. It can also improve monitoring of immunization programs.

Applications of geospatial technologies for immunization are often approached as simple solutions to system challenges without careful consideration of the greater ecosystem or planning for widespread adoption and sustainability [7]. Interventions are often deployed as pilot technology-focused projects without sustained resources or commitment to support the underlying enabling environment, human capacities, and governance systems that will contribute to a long-lasting impact on decision-making and health outcomes [8]. Gavi, the Vaccine Alliance, supports a systematic approach to understanding the range of geospatial data and technology implementation experiences to guide sustainable and effective systems and governance for improving immunization services that can reach every child with life-saving vaccines while strengthening primary health care systems [9]. Geospatial data and technology applications for immunization align with GAVI’s 2021-2025 strategy and the global Immunization Agenda 2030 strategy [9,10]. In order to provide life-saving services to children who default on the vaccination schedule and “zero-dose” children who have never received a vaccine, new geo-enabled approaches to planning and delivering services are needed to expand the reach of effective vaccination for all children.

## Theory of Change

**Figure 1:**
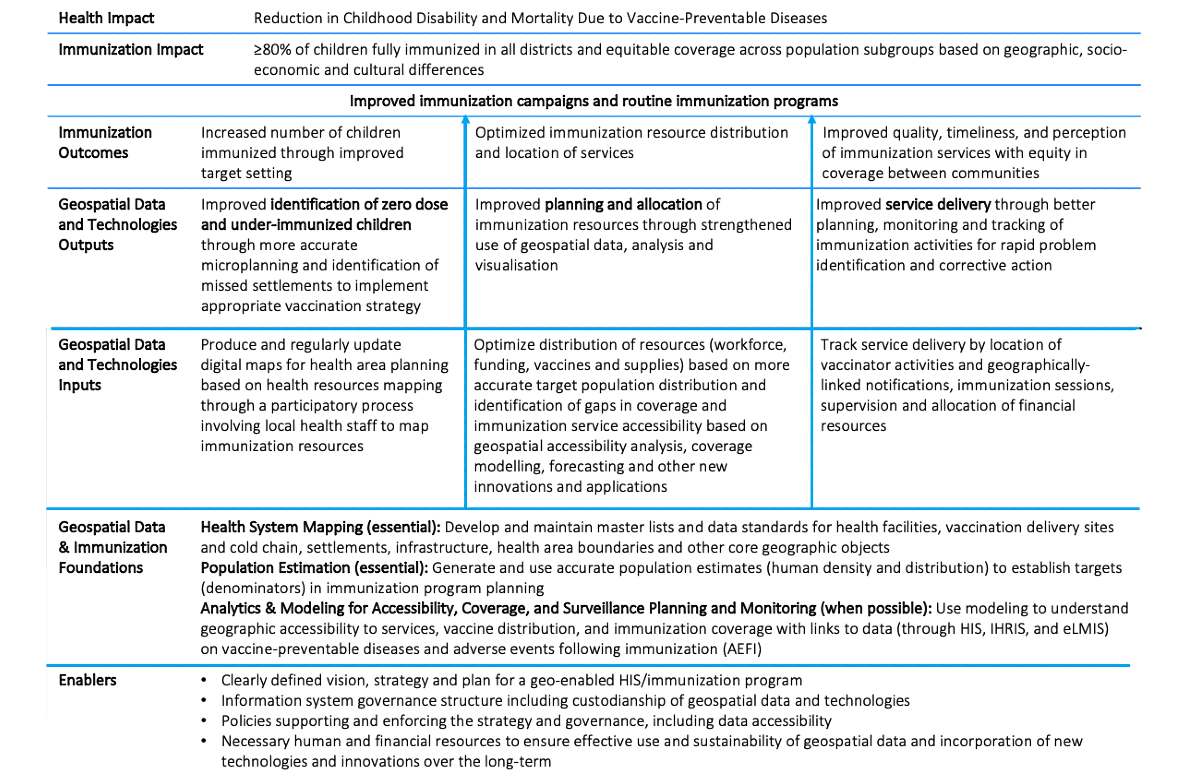
Theory of change for the use of geospatial technologies for immunization programing (originally published and adapted from [12]), with permission from Gavi, UNICEF, and HealthEnabled.

Complex interventions benefit from collaborative efforts to understand the underlying series of events and changes that will lead to the desired result [11]. A *theory of change* is a process and framework to help describe this causal pathway and to support critical thinking throughout the project design, implementation, and evaluation cycle [11]. *A theory of change for the use of geospatial technologies for immunization programming* describes the potential for geospatial technologies to contribute to real-world impacts by optimizing routine immunization program design, implementation, and monitoring to reach all children with immunization services (Figure 1). It was developed as part of a collaboration between GAVI and UNICEF (United Nations Children’s Emergency Fund) to review the state of evidence in the published and grey literature and through consultations with implementers, donors, immunization, and geospatial technology experts, as well as country-level implementation teams [12]. The theory of change is meant to guide future investment and planning of geospatial technologies and systems for immunization programs within a broader context of health system strengthening, to coordinate donor and partner collaboration, and optimize investments in foundations and systems for long-term sustainability and effective use of immunization data for decision-making.

## Evidence From Research and Implementation Experiences

### Overview

Geospatial data and technologies contribute to the following three interrelated immunization outcomes in the theory of change that together strengthen immunization campaigns and routine immunization program coverage and equity:

1. Increase the number of children immunized through improved target setting
2. Optimize immunization resource distribution and location of services
3. Improve the quality, timeliness, and perception of immunization services with equity in coverage between communities

These three outcome pillars are supported by foundations and enablers in the health system and a foundation of essential data that serve to guide the collection, management, and sustainable use of geospatial data and technologies for health. The theory of change is based on evidence and implementation experiences described for each of the three expected outcomes below.

### Increase the Number of Children Immunized Through Improved Target Setting

Despite years of improvements in global vaccination coverage and strengthening systems for service delivery, many children remain underimmunized or never come in contact with routine immunization programs [13]. Delivering life-saving immunization services to all children requires an enormous amount of coordination, planning, and resources; microplans are the local-level operational workplans used by immunization managers to systematically compile relevant local data, prioritize activities, maintain adequate stock, and find solutions to service delivery barriers [14]. UNICEF and the World Health Organization (WHO)’s Reach Every District (RED) strategy encourages the use of maps for local-level microplanning activities, which are traditionally hand-drawn sketches of the catchment area based on local knowledge [15]. These sketch maps are often not to scale; inaccurate or incomplete; and do not contain crucial information for microplanning such as distances, road conditions, or geographic barriers that may delay or discourage vaccinator teams from reaching remote areas during door-to-door campaign activities [3,16]. Health system data may contain overlapping borders; settlements that fall outside health boundaries; and inconsistencies in naming, spelling, and classification of service delivery units and settlements [2,4]. The planning tools and delivery strategies to reach all children with immunization services need to expand beyond the current methods to incorporate new digital tools that support local immunization managers to identify and reach areas that have been historically left off maps and microplans [17].

Children who have never received a vaccination can be clustered in settlements or neighborhoods, increasing their risk of contracting a vaccine-preventable disease without the benefit of herd immunity in their communities [18,19]. Due to a variety of socioeconomic and geographic barriers, these children are left “off the map” both literally and figuratively. Geospatial data and technologies for immunization programming can help identify these underreached communities by pinpointing the physical location of all settlements relative to the area’s immunization service delivery locations. Spatially accurate maps are created through a combination of satellite images and field-based data collection to georeference and validate landmarks, inhabited settlements, and infrastructure through a participatory process with district and local immunization managers. They are then used to plan and execute realistic action plans that include outreach activities. Microplans developed with geospatial technologies and data are a cost-effective way to identify settlements missed with traditional microplanning activities that rely on hand-drawn paper maps [2,5,6,16]. With more accurate and reliable information about the locations, characteristics, and number of settlements within their catchment area, managers can plan and prioritize their activities to vaccinate more children and monitor progress both from the local and central levels.

### Optimize Immunization Resource Distribution and Location of Services

Deciding how many vaccinators are needed for each catchment area, how many vaccines to send, and where to deploy fixed and outreach vaccination services depends on the number of people being served in each area, their distribution in the area, and the current unmet need for immunization services. The target population, or denominator, is often estimated from the most recent national census, adjusted each year by adding a fixed rate of growth [16]. Unfortunately, outdated census data, variation in growth rates, and population migration and mobility contribute to overestimation of the target population, leading to wasted resources, or underestimation with subsequent shortages and unvaccinated children [16,20,21]. Even with good population estimates, the location of settlements in relation to services measured by distance or travel time impact access and coverage. There is a relationship between complete and timely vaccination status and shorter distance or travel time to the nearest vaccination service, demonstrating how important the location of immunization services and geographic accessibility is for maximal immunization coverage [1,22-25]. To calculate unmet need, aggregate vaccination coverage data for the entire country or province can hide pockets of low coverage and settlements with unvaccinated children, leaving these communities vulnerable to vaccine-preventable diseases [26,27]. These data limitations impact immunization program planning and resource distribution, thereby preventing the timely delivery of life-saving vaccines to all children.

Tools and approaches that utilize geospatial technologies can help immunization managers make more targeted decisions for where and how to focus activities and resources. Precise estimates of population density and distribution for small geographic areas can be generated with a combination of satellite image data, statistical modelling, and sampled survey information to create accurate program targets for planning and monitoring purposes [28,29]. Population distribution estimates can be combined with spatial data on the location of vaccine service posts, road and transportation infrastructure, and geographic barriers to quantify the movement opportunity for people to reach existing services, inform new strategies and location of services, and prioritize outreach activities to remote communities [30,31]. Modelled vaccination coverage for small subnational units of measurement can be generated using multiple sources of data to identify pockets with low coverage and, when combined with data on other socioeconomic indicators, can help suggest solutions to overcome the social, gender-related, economic, geographic, or other factors that are preventing access to immunization services [32]. Improved granular data that is visualized to show geographic trends for local populations can help target delivery strategies and resources to increase immunization coverage in the areas that need it the most [33,34].

### Improve the Quality, Timeliness, and Perception of Immunization Services With Equity in Coverage Between Communities

A number of underlying factors contribute to nonvaccination—from service delivery challenges in the immunization program to community demand, including the caregiver’s perceived quality of immunization services, trust, and respect within the community [35,36]. Pockets of communities that do not receive quality and timely immunization services are susceptible to vaccine-preventable disease outbreaks. Measuring and monitoring these geographic and socioeconomic pockets of inequality is the first step toward promoting equality in coverage [37,38]. Timely and accurate data on program performance such as tracking supply and logistics, frequency of outreach services, and drop-out-rates can be used to improve the quality of services by providing entry points for supportive supervision, improve planning, identify problems, and initiate rapid corrective action for better overall service delivery [8,39]. Vaccine-preventable disease surveillance systems require rapid communication of data that facilitate feedback up and down the surveillance chain for coordinated and appropriate investigation and response [40]. In order to respond quickly to gaps and challenges, local and subnational immunization managers need to have the skills to use data that is collected accurately, transferred quickly, and presented in a way that can trigger action [8].

Mobile technologies and cellular networks provide opportunities to improve data collection, transfer, analysis, and use [41]. The combination of near real-time communication with automatic collection of accurate location data enables field-based teams of vaccinators to report on the number and location of doses delivered and any barriers encountered during immunization campaigns into an integrated dashboard where managers can monitor progress and respond appropriately to challenges and missed communities [42-45]. These daily reports of progress during campaign activities can help inform the next day’s strategy or provide evidence to extend or alter activities to reach all children in the target area [43-45]. For routine immunization services, supervisors can track the progress of mobile vaccination sessions as part of a geo-enabled digital microplan to identify and respond to missed settlements and improve monitoring of the microplan implementation [3,46]. The collection of geographic information linked to reports of suspected vaccine preventable diseases can facilitate rapid and coordinated action to prevent outbreaks, identify high-risk areas that need vaccination services, and facilitate risk-mapping to predict future outbreaks [40,47]. The transparent sharing of data can promote a common understanding of expectations and challenges between vaccinators and supervisors.

### Implementation Experiences: Myanmar Case Study

The current knowledge base shows that geospatial data and technology applications for immunization have the potential to stimulate programmatic improvements and increase immunization coverage. However, real-life examples of comprehensive and sustainable systems using geospatial data and technologies for immunization are rare. Myanmar provides an example of how the process of integrating geospatial data and technology for immunization microplanning validates the progression of incremental steps outlined in the theory of change.

In 2016, the national immunization program in Myanmar undertook a review as part of a health system commitment to creating a geo-enabled health information system. The assessment uncovered gaps in immunization coverage for children living in geographically and socially hard-to-reach communities, such as migrant worker settlements, remote villages, ethnic minority communities, and conflict-affected areas [48]. The local-level operational immunization workplans lacked reliable population information, and boundaries were out of date. This limited the ability of health workers to plan and undertake the daily logistics of immunization service delivery. In response to these gaps in coverage, the program took steps to support the microplanning process with geospatial data and technologies.

A phased pilot approach began in late 2017 in one township to begin building foundations, local capacity, and standard procedures and to demonstrate the benefits of using geospatial data and technologies for local-level immunization microplanning [48]. Subsequent expansion to a larger region in 2018 built on the foundations and lessons learned from the first pilot, as well as made improvements in the processes and implementation approach. Each expansion phase to a new area lasted 6 to 9 months to ensure that local capacity and systems were strengthened along the way.

The field implementation process created an up-to-date geo-referenced master list of facilities, settlements, and health area boundaries. A master list establishes a standardized, complete, up-to-date, and uniquely coded list of all features essential to the delivery of immunization services. Through this collaborative process, standard definitions were established for the geographic objects relevant to the microplanning process (eg, vaccination sites, facilities, and communities), and procedures were established for standard data collection. Every location where people lived, including temporary migrant settlements, were identified, defined, and included in the master list. Satellite images aided in settlement identification and catchment area delineation. Health workers were important stakeholders in the process to validate and review the maps and make necessary adjustments to their immunization microplans based on available transportation routes, distances, and geographic features in coordination with their supervisors. Online and printed maps showing accurate spatial relationships between key immunization assets and communities were produced and made available for national immunization program staff to plan vaccination campaigns and routine service delivery.

The interim results from Myanmar’s phased implementation approach include immediate effects of the collaborative process, map production, and distribution. With settlements and communities well defined, including characteristics and locations of temporary settlements, health workers were able to include these previously overlooked populations in their immunization microplans. The addition of missed settlements improved target population estimates, allowing for improvements in service delivery planning. The transparency and sharing of microplans and maps enabled supervisors to provide better support to health workers and encouraged accountability at all levels. Health officials were able to see the need for expanded health facility distribution with a clear visualization and accurate distances displayed in new microplanning maps.

These experiences validate the expected outputs for the integration of geospatial data and technologies in the theory of change (Table 1). Myanmar’s process of integrating geospatial data and technologies for immunization microplanning demonstrates how the complex challenge of delivering effective vaccinations to every child in countries with underlying health system challenges can benefit from these applications. Based on the implementation experiences in Myanmar’s program, it seems likely that continued expansion and improvements in the geo-enablement of their immunization program will lead to the desired immunization outcomes and overall expanded coverage as the theory of change suggests.

**Table 1:** Summary of geo-enabled microplanning implementation results from the Myanmar Central Expanded Program on Immunization.

Myanmar’s geo-enabled microplanning experiences	Corresponding geospatial data and technology theory of change output
Settlements that were previously missed are defined, identified, and included in the microplan Visualization of accurate geospatial relationships in catchment areas serve as a tool to plan vaccination sessions	Improved identification of zero-dose and underimmunized children through more accurate microplanning and identification of missed settlements to implement appropriate vaccination strategy
Target population denominator is closer to actual community density and distribution Standardized definitions and categorization of settlements and immunization resources help streamline planning process Visualization serves as an advocacy tool to demonstrate to senior health officials the need for improvements in the equitable distribution and allocation of resources	Improved planning and allocation of immunization resources through strengthened use of geospatial data, analysis, and visualization
Enhanced geo-enabled microplanning process encourages accountability of health workers and supervisors with transparency and shared expectations and service delivery plans	Improved service delivery through better planning, monitoring, and tracking of immunization activities for rapid problem identification and corrective action

Additional lessons from Myanmar’s experience reinforce the importance of the enabling environment and foundations in the theory of change, built on UNICEF’s guidelines and detailed approach to support the enabling environment for geospatial data and technologies in immunization programs [49]. The high-level commitment to transition to a geo-enabled national health information system in the Department of Public Health in Myanmar, with support and technical guidance from the WHO, GAVI, UNICEF, and the Health GeoLab Collaborative (a center of excellence for the Asia-Pacific region) laid a strong foundation of advocacy, governance, policies, and capacity for the management and use of geospatial data and technologies in the broader health sector [50]. The geo-enhanced microplanning process was further supported by the creation of common master lists for the geographic objects essential to the immunization program, an element recommended in the theory of change as an essential foundation to the sustained and effective use of geospatial data and technologies for immunization programs. A commitment to improving the supportive environment with a dedicated plan and resources to address needs and gaps in these enablers and foundations will promote the sustainable and effective use of geospatial data and technologies and the application of future geospatial innovations for immunization programs.

## Discussion: Applying the Theory of Change

As more immunization programs begin to incorporate geospatial data and technologies to help achieve and measure improvements in equitable immunization service delivery, the *theory of change for the use of geospatial technologies for immunization programming* can guide discussions, decision-making, and consensus building for investment, development, and coordination. The theory of change represents a thought process aimed at understanding the underlying sequence of events that can contribute to sustained and effective improvements and should be considered a roadmap that is subject to change, improvements, and fine-tuning as more country-level experiences bring insights into best practices and real-world challenges. The three pillars and supportive foundations and enablers can help initiate conversations and identify needs and gaps in country immunization programs to make sound decisions for short-term and long-term planning and contribute to improving the broader health system through shared geospatial data, technologies, and resources.

The theory of change may also serve as a framework for operational research and evaluations by suggesting quantifiable research objectives that will contribute to the evidence base and help clarify the relationships and determinants of effective application and use of geospatial data and technologies. The use of geospatial data and technologies within immunization programs can improve not only the systematic collection and use of quality and transparent data for programming but also for measuring improvements and incremental achievements throughout the project cycle.

The framework presented here is grounded in lessons from a handful of implementation experiences and existing evidence from the literature. As more countries gain practical experiences in integrating geospatial data and technologies into national immunization programs, best practices will suggest improvements to this theory of change and will help guide other programs on the incremental steps, foundations, planning, and budgeting recommendations that contribute to the sustainable integration of spatial data for immunization programming. A number of global and regional centers are developing and testing practical guidance and also providing technical support, resources, and training to help national programs apply geospatial data and technologies for immunization and other health systems [49,51,52].

## Conclusions

Effective data use will be necessary to make additional gains in global immunization coverage. Technology can help improve the collection, visualization, and use of data to detect and address inequalities in coverage [8,38]. However, the quality and value of immunization data ultimately depends on the people who are collecting, analyzing, and using the data, not just the technology they are using [8]. Geospatial data and technologies are a means to an end. They can strengthen data-driven decision-making if they are aligned with immunization outcomes in ways that address program needs and reinforce people’s confidence and trust in the resulting data products and analyses. Optimizing the deployment of immunization services to make them accessible for newly identified communities will pave the way for anchoring primary health care services in underserved areas. A focus on investing in and building sustainable and equitable health and immunization systems with strong leadership and capacity to use the geospatial tools and technologies that are appropriate for each country program will be critical for delivering life-saving vaccines to all children.

## Abbreviations

GAVI: Gavi, the Vaccine Alliance
RED: Reach Every District
UNICEF: United Nations Children’s Emergency Fund
WHO: World Health Organization
